# Applications of liquid chromatography-mass spectrometry based metabolomics in predictive and personalized medicine

**DOI:** 10.3389/fmolb.2022.1049016

**Published:** 2022-11-03

**Authors:** Juntuo Zhou, Lijun Zhong

**Affiliations:** ^1^ Beijing Boyuan Precision Medicine Co., Ltd., Beijing, China; ^2^ Center of Medical and Health Analysis, Peking University Health Science Center, Beijing, China

**Keywords:** metabolomics, liquid chromatography, mass spectrometry, predictive medicine, personalized medicine, COVID

## Abstract

Metabolomics is a fast-developing technique used in biomedical researches focusing on pathological mechanism illustration or novel biomarker development for diseases. The ability of simultaneously quantifying thousands of metabolites in samples makes metabolomics a promising technique in predictive or personalized medicine-oriented researches and applications. Liquid chromatography-mass spectrometry is the most widely employed analytical strategy for metabolomics. In this current mini-review, we provide a brief update on the recent developments and novel applications of LC-MS based metabolomics in the predictive and personalized medicine sector, such as early diagnosis, molecular phenotyping or prognostic evaluation. COVID-19 related metabolomic studies are also summarized. We also discuss the prospects of metabolomics in precision medicine-oriented researches, as well as critical issues that need to be addressed when employing metabolomic strategy in clinical applications.

## Introduction

Precision medicine approaches aim to improve prognosis and stratification of patients *via* utilizing knowledge of biological mechanism and biomarkers (Mao and Chen, 2022). Precision medicine-oriented researches and practices have been successfully applied in many healthcare sectors including tumors (Veninga and Voest, 2021; Mateo et al., 2022), respiratory diseases (Beitler et al., 2022), cardiovascular diseases (Weldy and Ashley, 2021), digestive tract diseases (Masoodi et al., 2021) and inflammatory diseases (Bieber, 2022; Mao and Chen, 2022). Applications of precision medicine can be further subdivided into several aspects, such as early detection, large-scale screening, molecular phenotyping and prognostic prediction, focusing on different stages of whole healthcare procedure. Many techniques and strategies have been employed in researches for precision medicine, such as molecular biology and omics-related analyses including genetics, transcriptomics, proteomics and metabolomics (Jacob et al., 2019), showing distinctive advantages and drawbacks.

**FIGURE 1:**
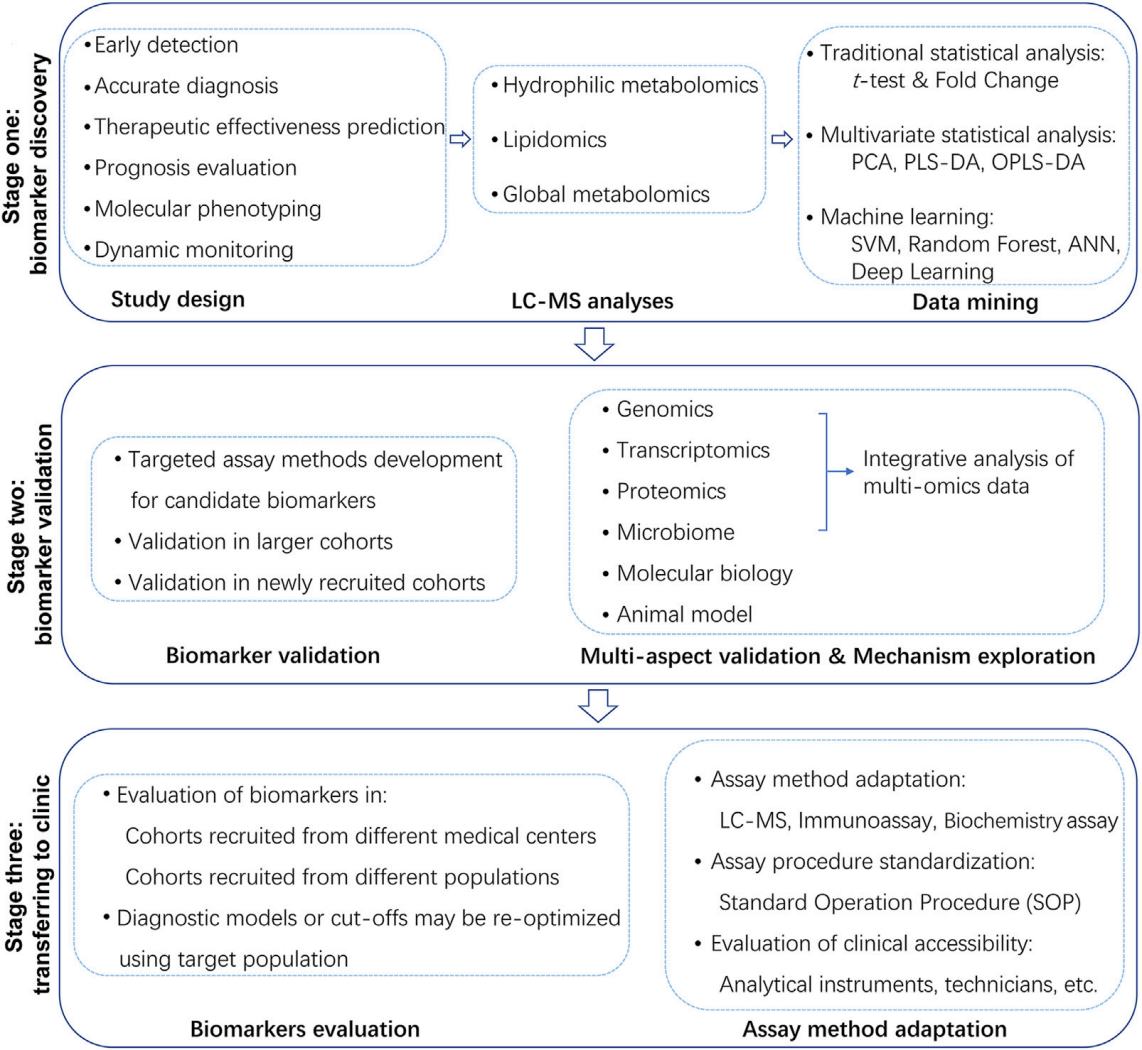
Flowchart of the typical steps of precision medicine-oriented metabolomic researches.

Metabolomics is a promising omics technique focusing on all metabolites presented in biological samples. It is a relatively lately-emerging and fast developing research area complementing genomics and proteomics, playing a more and more important role in researches aiming for biomarker discovery and personalized medicine (Seydel, 2021). Nuclear magnetic resonance (NMR), gas chromatography-mass spectrometry (GC-MS) and liquid chromatography-mass spectrometry (LC-MS) are common techniques used for metabolomic analyses (Jacob et al., 2019). LC-MS based metabolomics are becoming more preferred considering its wide metabolite coverage and high sensitivity (Zhou and Yin, 2016). Metabolomics can also be subdivided into polar metabolomics which focuses on polar metabolites and lipidomics which focuses on lipids (Heiles, 2021). The purpose of this mini-review is to provide a quick summary about the latest advances in precision medicine deriving from LC-MS based metabolomic researches (Table 1), providing insights into advantages, prospects as well as remaining challenges in this specific research sector. A flowchart presenting the typical steps of precision medicine oriented metabolomic researches is shown in Figure 1.

**TABLE 1:** Brief summary of LC-MS based metabolomic researches on precision medicine.

Research aim	Technique	Research subject	Sample type	Sample size	Results found in research	Experiments used to support metabolomic results	References
Diagnosis	Lipidomics	Oesophageal squamous cell carcinoma patients	Serum	525	A panel of 12 lipids for diagnosis	Tissue transcriptome	Yuan et al. (2021)
Diagnosis and early detection	Lipidomics	Pancreatic ductal adenocarcinoma patients	Serum	1,033	A panel of 17 lipid markers for diagnosis and early detection	Single-cell sequencing, proteomics and mass Spectrometry imaging	Wang et al. (2021)
Diagnosis and early detection	Lipidomics	Early-stage lung adenocarcinoma patients	Plasma	2,459	A panel of 9 lipid markers for diagnosis and early detection	Single-cell sequencing, proteomics and mass Spectrometry imaging	Wang et al. (2022)
Diagnosis	Lipidomics	Malignant brain gliomas patients	Plasma	2,002	A panel of 11 plasma lipids for diagnosis	Tissue transcriptome	Zhou et al. (2022)
Diagnosis	Metabolomics	Colorectal cancer or adenoma patients	Serum	440	Eight gut microbiome-associated serum metabolites for diagnosis	Metagenome sequencing of paired faecal samples	Chen et al. (2022)
Diagnosis	Metabolomics and NMR	Coronary artery disease patients	Serum	1,169	Metabolic deviations in coronary artery disease patients	Metagenome sequencing of paired faecal samples	Talmor-Barkan et al. (2022)
Prognostic prediction	Metabolomics and lipidomics	Acute traumatic brain injury patients	Serum	1,503	Panels of metabolites specifically associated with traumatic brain injury severity and patient outcomes	None	Talmor-Barkan et al. (2022)
Prognostic prediction	Metabolomics	Cirrhosis patients with acute kidney injury	Serum and urine	602	Urinary and serum metabolites used to predict acute kidney injury in inpatients with cirrhosis	None	Bajaj et al. (2021)
Prognostic prediction	Metabolomics	Hepatocellular carcinoma patients	Serum, liver tissue and stool	202	Metabolites associated with impaired liver function and poor survival	Validations using hepatocyte cell lines	Liu et al. (2022)
Molecular phenotyping	Metabolomics	Adults who had whole-genome sequencing data	Plasma	1,190	Associations between whole-genome sequencing and metabolic phenotyping for clinical assessments in adults	None	Hou et al. (2020)
Molecular phenotyping	Metabolomics	Cardiovascular disease patients	Plasma	2,330	Metabolic phenotype of young adults with cardiovascular risks	None	Murthy et al. (2020)
Molecular phenotyping	Metabolomics and lipidomics	Triple-negative breast cancer patients	Tissues	330	Linkage between metabolome to genomics & Three distinct metabolomic subgroups	Patient-derived organoid and xenograft models	Xiao et al. (2022)
Dynamic monitoring	Metabolomics and lipidomics	Healthy individuals	Plasma	100	Information on the stability in molecular profiles among healthy individuals over time	None	Tebani et al. (2020)
Dynamic monitoring	Metabolomics	Pregnant women	Plasma	30	A weekly characterization and a high-resolution landscape of the human pregnancy metabolome	None	Liang et al. (2020)
Molecular phenotyping	Metabolomics	COVID-19 patients	Plasma	139	A major immunological shift between mild and moderate infection	None	Su et al. (2020)
Diagnosis	Metabolomics and lipidomics	COVID-19 patients	Plasma and exosome	50	A panel of 10 plasma metabolites effectively distinguished COVID-19 patients from healthy controls	None	Song et al. (2020)
Prognostic prediction	Metabolomics	COVID-19 patients	Plasma	339	22 prognostic metabolites which predicts COVID-19 disease severity	A hamster model of COVID-19	Sindelar et al. (2021)
Diagnosis	Metabolomics	COVID-19 patients	Plasma	815	Molecules related to the disease’s pathophysiology or outcomes	None	Delafiori et al. (2021)
Molecular phenotyping	Metabolomics and lipidomics	COVID-19 patients	Serum	231	Molecular hallmarks of COVID-19, especially in those patients without comorbidities	None	Wu et al. (2021)

### Metabolomics for early detection, large-scale screening or diagnosis

Diagnostic biomarker development is one typical application of metabolomic studies. Reliable diagnosis at early stage of diseases usually lead to improved treatment efficacy and better outcomes (Crosby et al., 2022). In general, without typical symptoms at early-stage, diseases are detected and diagnosed by endoscopy, biopsy or imaging examination, which are usually invasive, costly or highly subjective. To address this problem, liquid biopsy-based metabolomic analyses are considered as relatively non-invasive and accessible diagnostic methods. Metabolomic studies have made great progresses recently in tumor diagnosis. Yuan et al. (2021) combined serum lipidomics to reveal lipid biomarkers for detection of oesophageal squamous cell carcinoma, and a panel of 12 lipids were finally found for diagnostic purpose. Wang et al. (2021) employed metabolomics in search for blood diagnostic biomarkers of pancreatic ductal adenocarcinoma and early-stage lung adenocarcinoma (Wang et al., 2022), revealing 17 and 9 potential lipid markers in blood separately. Zhou et al. (2022) applied untargeted lipidomics in search for biomarkers for malignant brain gliomas, finding a panel of 11 plasma lipids for diagnosis. Chen et al. (2022) explored serum metabolome of patients with colorectal cancer or adenoma, and successfully found eight gut microbiome-associated serum metabolites that could accurately discriminate patients from normal individuals. Talmor-Barkan et al. (2022) performed serum metabolomics for 199 patients with acute coronary syndrome, revealing personalized risk factors for coronary artery disease. These researches focused on blood or urine-derived samples for biomarker development, taking advantage of the non-invasive and side effect-free features of blood tests, thus have great potential to be employed for large-scale screening in populations with high risks or in regular physical examination for normal population. Of course, at current stage the biomarkers found in these researches cannot be used in actual clinical practice until further validation and evaluation with strict clinical trials are finished.

### Metabolomics for prediction of therapeutic effectiveness or prognosis

Personalized treatment is an important sector in precision medicine. In clinical practice, considering the individual difference of patients, the therapeutic effectiveness and response rates of a given drug or treatment usually varies among patients. So, it is important and valuable to predict therapeutic effectiveness or prognosis as early as possible for each individual. Thomas et al. (2022) used metabolomics to describe the serum metabolome and lipidome associated with acute traumatic brain injury as well as its relationship with injury severity and patient outcome. Bajaj et al. (2021) performed serum and urine metabolomics to reveal predictive markers for acute kidney injury in patients with cirrhosis, potentially facilitating earlier initiation of renoprotective measures. Liu et al. (2022) performed integrative metabolomic analyses of portal venous blood, liver tissues and stool samples of patients with hepatocellular carcinoma, finding several novel metabolites associated with impaired liver function and poor survival. Development of predictive biomarkers for therapeutic effectiveness or prognosis can provide valuable information to doctors at early disease stages, giving doctors precaution to choose best treatment strategies or take actions earlier, making treatments more efficient and targeting.

### Metabolomics for molecular phenotyping or dynamic monitoring

Due to the diversity of metabolite expressive profile of each individual person, metabolome can be seen as a character to classify individuals with disease risks. Compared with traditional phenotyping strategy using pathological or genetic results, molecular phenotyping with metabolome, which can provide a novel insight into disease subtypes at molecular level, is emerging as a promising research strategy. Hou et al. (2020) used metabolomics to phenotype a cohort of 1,190 adults, facilitating the identification and clinical assessment of adults with genetic disorders. Murthy et al. (2020) studied 2,330 young adults to identify metabolite profiles associated with an adverse cardiovascular disease phenome, finding that metabolite-based health scores can predict higher hazard of future adverse cardiovascular disease, and proving efforts to include precision measures of metabolic health is important to interrupt adverse cardiovascular disease at its earliest stage. Xiao et al. (2022) profiled the metabolome in triple-negative breast cancer tissues samples to classify breast cancer into three distinct metabolomic subgroups which have corresponding novel therapeutic targets, promoting precision treatment of triple-negative breast cancer. Another application aspect of metabolomics is to monitor molecular profiles among healthy individuals over time. Tebani et al. (2020) performed metabolomics-included multi-omics analyses using a longitudinal wellness cohort with 100 healthy individuals, finding that comprehensive omics profiling in a longitudinal manner as a way to monitor body status is vital for precision medicine. Liang et al. (2020) analyzed the metabolome of weekly blood samples collected from 30 pregnant women to define a high-resolution temporal profile of metabolites during healthy pregnancy, providing a high-resolution molecular landscape for understanding pregnancy and potential clinical applications. Metabolomics-based molecular phenotyping strategy can help us better understand individual differences at molecular level, providing important information and insight for precision medicine practice. Also, molecular phenotyping or dynamic monitoring for diseases or physiological processes at metabolic level are relatively new research aspects which have no other gold standard methods, making it a promising research strategy.

### Metabolomics facilitates studies of COVID-19

Metabolomic analyses have also played important roles in infectious disease-related researches. In recent years, the coronavirus disease 2019 (COVID-19) pandemic has presented an unprecedented threat to global healthcare system (Rubin, 2021; Peeling et al., 2022). Metabolomic techniques have played an important role in studying COVID-19 to achieve more accurate treatments. Su et al. (2020) employed metabolomics as part of their multi-omics analyses to resolve a sharp disease-state shift between mild and moderate COVID patients, suggesting that moderate disease state may provide the most effective setting for therapeutic intervention. Song et al. (2020) utilized a combination of targeted and untargeted metabolomics to analyze the plasma lipidome and metabolome in COVID patients and healthy controls, finding molecules correlated with virus infection and disease severity. Sindelar et al. (2021) and Delafiori et al. (2021) employed metabolomic analysis of patient plasma to successfully construct diagnostic models that predict COVID-19 infection risk and disease severity, and the models are applicable to most hospitals around the world. Wu et al. (2021) integrated metabolomics to multi-omics analyses to provide a landscape for COVID-19 patients without comorbidities. In summary, the implement of metabolomic techniques in COVID related researches has provided valuable clues regarding pathological mechanisms, diagnostic biomarkers or novel therapeutic strategies for COVID-19.

## Discussion

### The advantages of LC-MS based metabolomics in precision medicine

Among the strategies employed in precision medicine-oriented researches, LC-MS based metabolomic technique has some advantages. One important feature is its broad metabolite coverage ranging from polar metabolite to non-polar lipids. Combing the deep coverage and high sensitivity (limit of detection usually reaches ng/mL in biological samples), metabolomics enables the analyses of low-level molecules in samples and make it practical to detect disease at an early disease stage. Second, LC-MS based diagnostic strategies can achieve high-throughput analyses by taking advantage of fast separation performance of ultra-high performance liquid chromatography (UPLC) and fast scan speed of triple quadrupole mass spectrometry (QQQ-MS). It has potential to analyze tens to hundreds of metabolites in less than half an hour of analytical time, making it suitable for large-scale screening in a large population, facilitating timely identification of diseases during a regular physical examination. Taking together, LC-MS techniques can be employed in precision medicine-oriented researches in two stages with different focuses. Untargeted metabolomics, which is usually performed on high-resolution MS instruments such as Q-TOF and Q-Orbitrap, is suitable for biomarker discovery stage taking advantage of its wide metabolite coverage which could detect thousands of molecules in samples. After data mining, biomarker panels, which usually consisted of several to dozens of metabolites, are revealed. Next, LC-MS based targeted quantification assay, which is usually performed on low-resolution triple quadrupole MS instruments (QQQ-MS), can then be used for accurate and repeatable quantification of these biomarker panels to take advantage of its high-throughput and accuracy. Third, as biomarkers, metabolites can reflect the body status more accurately rather than genomics and transcriptomes because metabolome can most directly reflect the underlying biochemical activities of certain cells and tissues in human bodies, thus can most accurately represent the molecular phenotype of diseases (Piano and Cardenas, 2021), presenting “what has happened in bodies” rather than “what will possibly happen” (Seydel, 2021). In this regard, measuring and monitoring the metabolome can provide key information for pathophysiological mechanisms and provide an essential piece of jigsaw for precision medicine. Up to days, in the area of precision medicine, metabolomic analyses are relatively less widely used compared to gene or protein-based analyses, but as the rapid development of LC-MS techniques and data mining strategies, metabolomics is emerging as a preferable and promising strategy, and will greatly facilitate precision medicine in clinical practice.

### Data mining procedures for metabolomics

Apart from performance of LC and MS instruments, data mining procedure can also play an important role in metabolomic researches. Considering the complexity of data generated by untargeted metabolomics, a suitable and reliable data mining strategy is key to retrieve robust conclusions. Statistical analyses such as fold-change, *t*-test and ANOVA, multivariant statistical analyses such as Principal Component Analysis (PCA), Partial Least Squares Discriminant Analysis (PLS-DA) and Orthogonal Partial Least Squares-Discriminant Analysis (OPLS-DA), are traditional data processing procedures. In recent years, as development in algorithms, machine learning is becoming more and more widely employed in data mining procedures of metabolomics to retrieve biomarker panels for better performance (Reel et al., 2021; Li et al., 2022). For example, researchers have used support vector machine (SVM)-based machine learning models to illustrate diagnostic biomarkers in blood for oesophageal cancer (Yuan et al., 2021), pancreatic cancer (Wang et al., 2021), lung cancer (Wang et al., 2022) and brain glioma (Zhou et al., 2022). The SVM-based algorithm can rank all potential biomarkers according to their weight in the model, making it practical to optimize the candidates for a biomarker panel. Researchers have also employed deep learning algorithms to estimate the weighted detection probability of peaks in LC-MS data, or eliminate false-positive peaks without reducing the true positive rate (Woldegebriel and Derks, 2017; Melnikov et al., 2020). In all, the employment of AI algorithms in metabolomic data mining has improved the efficiency and performance of data processing. But attentions must be paid when choosing data mining procedures because performance of algorithms may vary according to data matrix. For example, SVM is an ideal classifier for two-class classification, especially for a training dataset which consists a small sample size (Zhou et al., 2022). Thus, choosing a suitable data mining strategy for each data matrix is important for achieving a high-quality result. What’s more, considering the complexity of untargeted metabolomic data, results generated by different data pre-processing and data mining strategies (such as different normalization or statistical algorithms) usually differ in some way, so validation of biomarkers by different assay methods (such as targeted quantification, ELISA or biochemistry assay) is necessary before drawing a conclusion.

### Important issues need to be concerned when employing metabolomics in precision medicine

There are some issues need to be concerned when employing metabolomics in precision medicine. First, study design is important in metabolomic analyses aiming to develop biomarkers for diseases. Metabolome is fast changing and sensitive to non-disease factors such as environment, population diversity, diet, drug treatment, personal lifestyle, sample collection and storage procedures (Després, 2020), thus, a rigorous study design is important to exclude influences of non-disease factors. What’s more, a large sample size is also needed for patients-derived metabolomic researches considering that metabolite profiles show high variation among individuals, and the normal levels and diagnostic cutoffs of certain biomarkers may vary among different subject cohorts or populations (Tebani et al., 2020). That is also why after biomarker discovery stage, large-scale validations at different medical centers or populations are further needed to validate the diagnostic abilities before candidate biomarkers can be finally used to guide clinical treatment protocols, and is also why diagnostic models sometimes need to be optimized for a given targeted cohort or population for each application (Wenk and Choi, 2022). Usually, to conclude a reliable conclusion, thousands of participants are needed for researches covering discover and validation stages.

Mechanism evidence is also necessary and supportive for a reliable metabolomic research aiming to facilitate clinical practice. Metabolome of blood sample is the collection of metabolites generated from cells in the entire body, reflecting the sum of intercellular and extracellular biochemical reactions, so it should be verified that the change of candidate biomarkers in blood is caused by targeted abnormal tissues rather than other normal tissues or organs. Mechanism evidences can be acquired *via* molecular biology experiments, while genomics, transcriptomics and proteomics are also ideal complements to metabolomics. As researches conducted by Wang and colleagues, they performed transcriptomics or *in situ* imaging using tumor tissues to illustrate the underlying mechanism, validating that the dysregulated lipids found in blood was caused by dysregulated metabolic status in corresponding tumor tissues (Wang et al., 2022). In the metabolomic research conducted by Chen et al. (2022) and Talmor-Barkan et al. (2022), metagenome sequencing-based gut microbiome analyses of paired fecal samples were used to illustrate the origin of the dysregulated metabolite biomarkers in serum, proving the true association between the found diagnostic biomarkers and the diseases. Xiao et al. (2022) performed experiments on patient-derived organoid and xenograft models to illustrate the underlying mechanism of the found dysregulated metabolome in triple-negative breast cancer. Sindelar et al. (2021) validated the dysregulated metabolites in COVID patients in a hamster model of COVID-19, further verifying the reliability of the found biomarkers. In all, extra experimental evidence is important to improve the reliability of metabolomic results as well as to better understand the mechanism underlying metabolomic changes.

### The role of metabolomics in multi-omic analyses

Metabolome is one of the omics comprising the biological system of human body. Each omics data plays an unique and necessary role in the fully functional system, it is not possible to use metabolome along to fully illustrate the underlying mechanism of certain diseases. Thus, combining metabolomics with genomic, transcriptomic or proteomic studies will result in a significantly improved understanding of disease mechanisms and the pathophysiology of target clinical phenotype. This multi-omics approach will represent a major step forward toward achieving precision medical care. What’s more, metabolome is a highly dynamic system, metabolite levels depend on the enzymes that consume and produce them. The systemic properties of a metabolic network rely on fluxes (the actual dynamic picture of the phenotype, thus a unique phenotypic signature) which, beyond metabolites and enzymes, also depend on regulatory feedback which cannot be fully accounted for only by metabolomics data. So, integrating metabolomic data into other omics data is important for illustrating pathological mechanisms to facilitate precision medicine. At present, integrating metabolomic data with other omics is not as easy as data integration between genomics, transcriptomics and proteomics, because there is not a consistent one-to-one match between metabolite and genes. Efforts are still needed to improve bioinformatic algorithms for integrating metabolome with other omics.

### Challenges for metabolomics in clinical application

The strategy of employing metabolomics in precision medicine still has some challenges. The first one is the non-repeatability of found biomarkers and non-reproducibility of many studies. The untargeted metabolomic results, which are usually relative or semi-absolute quantitative results, may vary on different LC-MS instrument platforms as well as in different labs. The standardized quantification result of biomarkers is essential to expand the analytical conclusion among different labs and medical centers. To achieve this goal, the LC-MRM-MS assay method, which employed standard curve and isotopic internal standards to realize absolute quantification, is an ideal choice. Lack of external validation using expanded cohorts may also results to unreliable conclusions. The second challenge is the specificity of found biomarkers and interpretability of predictive models. Due to the study design of metabolomics which usually compare only one certain kind of disease and corresponding control group, the found dysregulated metabolite may also change in other disease conditions, decreasing the diagnostic reliability. For accurate and robust diagnosis, we need biomarkers which are highly relevant to the targeted disease and resistant to influences of non-disease factors such as environment and diet. Thus, strong clinical relevance of biomarkers and consistent predictive performances of diagnostic models are necessary when transferring to clinical applications. But analyzing samples of every other disease to verify the specificity of the found biomarkers is not practical. In actual practice, we could only try to analyze as much sample as we could collect to verify the biomarkers’ specificity and lower the possibility of misdiagnosis. The third challenge is translating scientific research to clinical applications. Even though there have been a great number of high-quality scientific researches which have used metabolomics in precision medicine, only few have been successfully applied in clinical decision making, showing a gap between scientific research and clinical practice (Trifonova et al., 2021). This is partly because of the different focuses between scientific research labs and clinical applications. For scientific research, biomarker screening result depends on statistical analyses which presents differences among different statistical strategies. For example, raw *p*-values usually present more dysregulated biomarkers but may include more false positives. Multiple testing generated parameters such as q-values (adjusted *p*-value generated by Bonferroni or FDR algorithms) can lower the false positives but false negatives may occur. What’s more, a significant difference determined by statistical analyses does not equal to ideal biomarkers for clinical practice. For example, a metabolite with a *p*-value less than 0.05 can be considered significantly changed between groups even though its levels largely overlap between disease and control groups. But in clinical practice, if we cannot conclude a clear cutoff value for a biomarker’s level (which needs that a biomarker’s levels would separate clearly between disease and control groups), it will be difficult to use it for accurate diagnosis. Besides, to successfully transfer an assay method from a scientific lab to clinical labs, a standard operating procedure (SOP), standardized reagents and chemicals are essential. LC-MS instrument accessibility is also an issue, as well as the capability of clinical lab technicians considering that operating and maintaining LC-MS instruments need expertise and experience.

## Summary

Metabolomics has great prospect in the area of precision medicine. With the popularization of LC-MS instruments in hospitals and clinics, metabolomic researches will reveal novel biomarkers of diagnostic, prognostic or therapeutic value, facilitating clinical practices ranging from pre-hospital situations (early detection and large-scale screening) to post-hospital situations (diagnosis, subtyping, therapeutic effectiveness prediction and prognostic prediction) and significantly improving efficiency of doctors and satisfactory of patients. What’s more, as technological advances in the future, more cutting-edge metabolomic technique such as metabolite imaging and single-cell metabolomics (Seydel, 2021) can further facilitate the application of metabolomics in precision medicine.
